# Integrating artificial intelligence into healthcare systems: more than just the algorithm

**DOI:** 10.1038/s41746-024-01066-z

**Published:** 2024-03-01

**Authors:** Jethro C. C. Kwong, Grace C. Nickel, Serena C. Y. Wang, Joseph C. Kvedar

**Affiliations:** 1 Division of Urology, Department of Surgery, University of Toronto, Toronto, ON Canada; 2 Temerty Centre for AI Research and Education in Medicine, University of Toronto, Toronto, ON Canada; 3 Harvard Medical School, Boston, MA USA

**Keywords:** Health care, Diseases

## Abstract

Boussina et al. recently evaluated a deep learning sepsis prediction model (COMPOSER) in a prospective before-and-after quasi-experimental study within two emergency departments at UC San Diego Health, tracking outcomes before and after deployment. Over the five-month implementation period, they reported a 17% relative reduction in in-hospital sepsis mortality and a 10% relative increase in sepsis bundle compliance. This editorial discusses the importance of shifting the focus towards evaluating clinically relevant outcomes, such as mortality reduction or quality-of-life improvements, when adopting artificial intelligence (AI) tools. We also explore the ecosystem vital for AI algorithms to succeed in the clinical setting, from interoperability standards and infrastructure to dashboards and action plans. Finally, we suggest that algorithms may eventually fail due to the human nature of healthcare, advocating for the need for continuous monitoring systems to ensure the adaptability of these tools in the ever-evolving healthcare landscape.

## Introduction

Despite the rapid growth of artificial intelligence (AI) applications in healthcare, few models have progressed beyond retrospective development or validation, creating what is commonly called the “AI chasm”^1^. Among the subset of models that have moved into randomized controlled trials, even fewer have demonstrated clinically meaningful benefits^2^. This reality is a sobering reminder that translating AI algorithms from in silico environments to real-world clinical settings remains a formidable challenge. Possible reasons for this translational gap may be attributed to a high risk of bias during model development or dataset shifts during prospective validation^3,4^.

One of the conditions that has been extensively studied within the AI community is sepsis, life-threatening organ dysfunction due to infection, and a leading cause of morbidity and mortality worldwide^5^. Early identification of sepsis is paramount, as it enables timely administration of antibiotics and other life-saving measures. Therefore, the challenge and importance of early sepsis detection has catalyzed the development of several predictive algorithms across various clinical settings, including the emergency department (ED), inpatient ward, and intensive care unit (ICU)^6^. However, model evaluation concerning real-world patient outcomes has remained limited.

In this context, Boussina and colleagues should be congratulated for their efforts to demonstrate significant improvements in patient outcomes after implementing their AI algorithm^7^. The authors previously developed COMPOSER (COnformal Multidimensional Prediction Of SEpsis Risk)^8^. This deep learning model imports routine clinical information from electronic health records (EHR) using retrospective data to predict sepsis (based on the current Sepsis-3 criteria). In the present study, they first conducted a “silent mode trial,” evaluating their model on prospective patients in real-time while end-users were blinded to predictions. Next, they performed an implementation experiment that tracked patient outcomes before and after the deployment of COMPOSER. Their approach was well-aligned with the three-stage translational pathway for AI, which comprises (1) exploratory model development, (2) a silent trial, and (3) prospective clinical evaluation^9,10^. Here, the authors found that using COMPOSER within two EDs at UC San Diego (UCSD) Health was associated with a 17% relative reduction in in-hospital mortality and a 10% increase in sepsis bundle compliance. Sepsis bundles may vary across institutions but are generally composed of actions such as obtaining blood cultures before administering antibiotics, measuring lactate at defined time intervals, and administering fluids within three hours of presentation.

### More than just the AI algorithm

Importantly, this study offers valuable insights into the ecosystem required for AI algorithms to perform well in the clinical setting in the United States. COMPOSER was directly embedded into the clinical workflow, following similar principles described by Sendak et al.^11^. A nurse-facing Best Practice Advisory (BPA) (i.e., a reminder/warning) presenting the COMPOSER sepsis risk score alongside top predictive features was integrated into the EHR. This was an essential step towards addressing the critical need for explainability among clinical end-users^12^. A standardized set of responses to the BPA was devised with multidisciplinary input. This broad stakeholder engagement was likely vital to achieving a remarkable degree of buy-in among nurses, with only 5.9% of sepsis alerts dismissed over the five-month intervention period. Furthermore, the BPA enhanced communication between nurses and physicians and expedited time-to-antibiotics—a plausible mechanism for the observed reduction in mortality. Finally, the study team implemented robust systems to continuously monitor data quality and model performance, prompting model retraining if performance fell below predefined thresholds. This approach ensures the sustained effectiveness and adaptability of COMPOSER over time.

As evident in that study, scaling AI algorithms within healthcare systems requires substantial resources, infrastructure, expertise, and adequate endorsement at the clinical end-user, departmental, and institutional levels. Such an ecosystem may be challenging outside of academic settings or within single-payer healthcare systems. Therefore, the costs and benefits of these AI algorithms should be carefully considered through health technology assessments because their incremental advantages may not justify the steep costs required to implement and maintain such technologies. Table 1 outlines key considerations for hospital leadership as they navigate implementing these algorithms within their institutions.

**Table 1:** Considerations for implementing AI algorithms into healthcare systems

Theme	Key considerations	How each issue was addressed by Boussina et al.^7^
Data	Are the data needed for the algorithm readily available and in an extractable format?	COMPOSER routinely collected clinical information, including laboratory and vital signs. Data elements were extracted via FHIR standards.
Infrastructure	Can relevant data be extracted in real time?	Data were extracted at hourly intervals to ensure availability for prediction.
	Are there adequate infrastructure and computing resources available to host a cloud-based analytics and storage platform?	The platform was hosted via Amazon Web Services.
Interface	How will the clinical team be made aware of these predictions (i.e., is a custom dashboard integrated into the electronic health record required)?	Predictions were integrated into an Epic flowsheet via an HL7v2 outbound message. A Best Practice Advisory was triggered for patients at high risk of developing sepsis.
	How can the clinical team understand how the algorithm made this prediction (i.e., model explainability)?	A relevance score was generated for each feature, which measured the gradient of the risk score with respect to all input features multiplied by the input features. The features with the highest positive relevance scores were displayed in the flowsheet.
End-users	Which clinical team member(s) are most appropriate to receive the risk prediction?	Nurses were chosen to receive the alert as they cared for a specific roster of patients and frequently opened their patients’ charts.
	What is their level of trust in the AI algorithm?	A multidisciplinary team was created to guide implementation. Nurses were surveyed to identify their needs. Regular feedback and educational sessions on COMPOSER were provided to nurses during the implementation phase.
	What is the risk of alert fatigue, burnout, or a decrease in algorithmic compliance over time?	Although end-user surveys were not reported, the non-compliance rate was low (5.9%) and remained stable throughout the implementation period.
Clinical context	Can a standardized action plan be implemented based on the risk prediction?	Nurses responded to the BPA by choosing one of three options: (1) indicating no suspicion of infection, (2) confirming ongoing sepsis treatment or workup, or (3) notifying the physician.
	Are there sufficient hospital resources (e.g., inpatient beds, operating room staff, or specific interventions) available for the clinical team to act on the risk prediction?	The clinical team could initiate antibiotics and fluids or order additional investigations in response to the BPA.
Monitoring	How will clinical end-users or hospital leadership know whether the model continues to perform well over time?	A data quality dashboard was developed to ensure input feature values fell within pre-specified limits. COMPOSER was monitored by measuring sensitivity and positive predictive value biweekly.
	What happens if the algorithm’s performance degrades over time (i.e., is there adequate infrastructure and expertise to retrain the algorithm)?	A Predetermined Change Control Plan was established to trigger retraining of COMPOSER if its performance fell below pre-specified thresholds.
	What happens if the compliance of clinical end-users degrades over time?	Although not specified, the authors acknowledged the importance of continuous education to optimize human-AI collaboration.

Examples of how these issues were addressed by Boussina et al. are provided

### Healthcare is only human

AI algorithms tend to excel in controlled environments, where only specific predictive features may influence the clinical outcome. However, patients’ and providers’ inherently human nature introduces numerous challenges, causing even the most robust AI models to degrade over time. Diversity in patient characteristics, disease presentations, practice patterns, and evolving treatment paradigms contribute to the potential failure of algorithms post-deployment^4^. Indeed, Boussina et al. highlight some of these challenges in their study. Despite a reported reduction in sepsis-related mortality, this benefit was only observed in one of the two hospitals. The lack of clinical improvement at their quaternary site may be attributed to differences in patient comorbidities, where even timely interventions may not be sufficient. In addition, the evaluation of COMPOSER was limited to the ED setting at UCSD thus, its generalizability in other clinical environments or institutions remains unknown. Similar concerns have been raised regarding the Epic Sepsis Model, which was found to have much lower performance and high false positive rates during external validation^13^. Lastly, clinical end-users may have been influenced by their awareness of being observed (i.e., Hawthorne effect) during the five-month implementation period, and their compliance with the BPA may diminish over time. These limitations emphasize the need for an AI ecosystem to support algorithms and enable them to adapt as healthcare continuously evolves.

## Conclusion

AI can only be successful in healthcare systems if their predictions are available at the right time and place. Algorithms, while critical, cannot function in isolation – they must be paired with dedicated infrastructure, resources, and personnel trained to act on their predictions. Processes must also be in place to enable algorithms to adapt when their predictions degrade over time due to the evolving healthcare landscape. Furthermore, AI researchers should shift the focus from measuring just performance metrics such as accuracy towards meaningful improvements in individual patient outcomes while balancing the potentially steep costs of technological innovation. As a healthcare and AI community, we have a responsibility to deliver on these clinically relevant metrics, and researchers and journals alike should be encouraged to prioritize such studies.
